# Identifying and tracking simulated synaptic inputs from neuronal firing: insights from in vitro experiments

**DOI:** 10.1186/1471-2202-16-S1-P239

**Published:** 2015-12-18

**Authors:** Maxim Volgushev, Vladimir Ilin, Ian H Stevenson

**Affiliations:** 1 Department of Psychology, University of Connecticut, Storrs, CT, USA; 2 Department of Biomedical Engineering, University of Connecticut, Storrs, CT, USA

## 

Accurately describing synaptic interactions between neurons and how interactions change over time are key challenges for systems neuroscience. Although intracellular electrophysiology is a powerful tool for studying synaptic integration and plasticity, it is limited by the small number of neurons that can be recorded simultaneously in vitro and by the technical difficulty of intracellular recording in vivo. One way around these difficulties may be to use large-scale extracellular recording of spike trains and apply statistical methods to model and infer functional connections between neurons. These techniques have the potential to reveal large-scale connectivity structure based on the spike timing alone. However, the interpretation of functional connectivity is often approximate, since only a small fraction of presynaptic inputs are typically observed.

Here we use in vitro current injection in layer 2/3 pyramidal neurons to validate methods for inferring functional connectivity in a setting where input to the neuron is controlled. In experiments with partially-defined input we inject a single simulated input with known amplitude on a background of fluctuating noise. In a fully-defined input paradigm we then control the synaptic weights and timing of many (1024) simulated presynaptic neurons. By analyzing the firing of neurons in response to these artificial inputs, we ask 1) How does functional connectivity inferred from spikes relate to simulated synaptic input? and 2) What are the limitations of connectivity inference?

**Figure 1:**
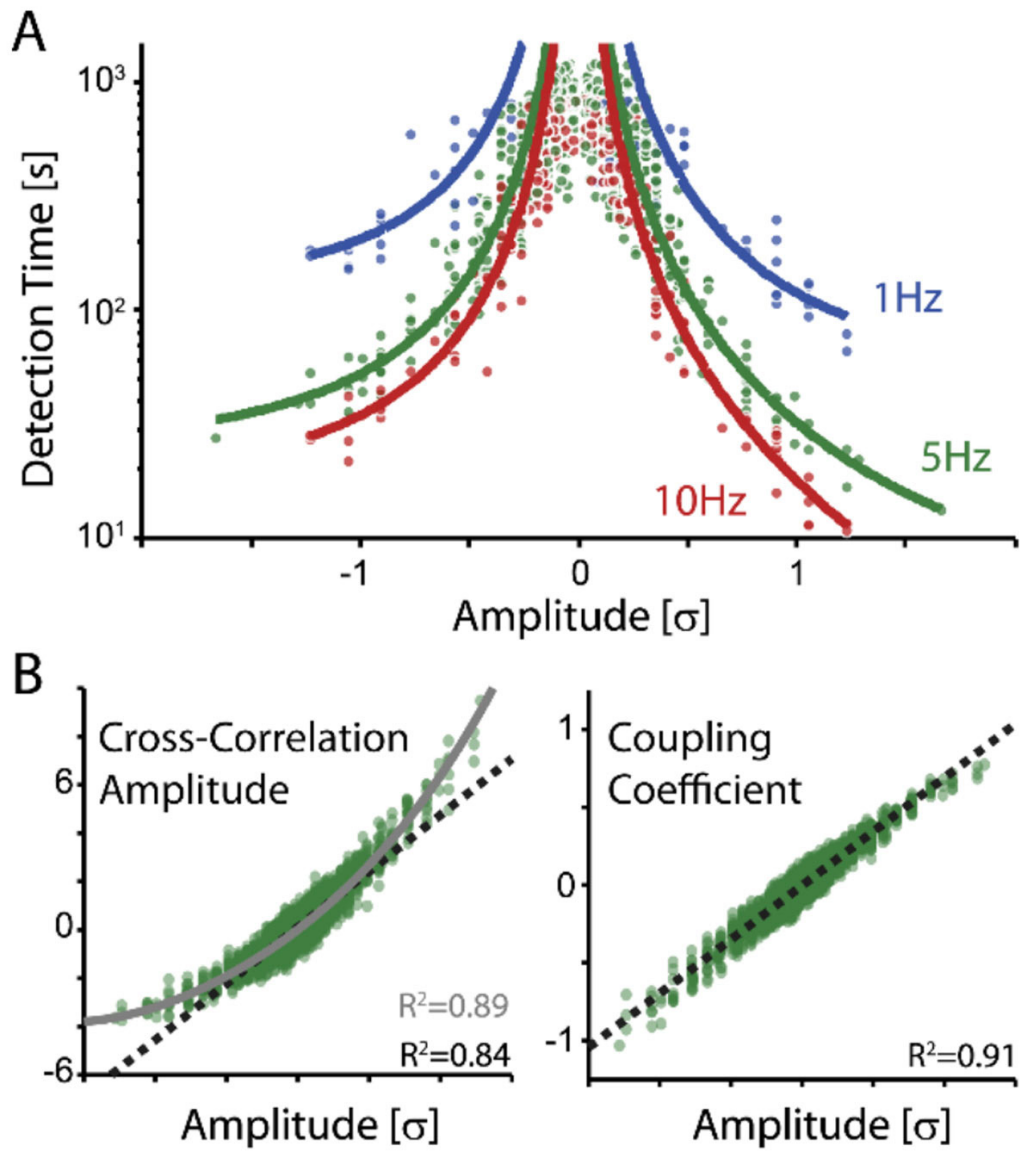
**A) Detectability of synaptic inputs of different strengths in a fully-defined input paradigm (data for N = 4 Layer 2/3 pyramidal cells) for 1Hz, 5Hz, and 10Hz post-synaptic firing rates**. Note that detection of inputs improves with higher firing rates and that inhibitory inputs require longer recording periods than detection of excitatory inputs. Detection time for inputs of different amplitudes drops as approximately c/x2. B, left) Ground truth PSC amplitude vs the amplitude of the cross-correlation (25ms window following pre-synaptic spikes) for 5Hz post-synaptic spiking. B, right) Ground truth PSC amplitude vs the model-based coupling coefficient. Note that model-based coefficients more accurately reconstruct the true amplitudes. Dashed lines denote a linear fit, gray line denotes a 4th-order polynomial fit to account for the nonlinear relationship between PSC amplitude and correlation coefficients.

Using likelihood-based models of functional connectivity, we find that individual current-based synaptic inputs are detectable over a broad range of amplitudes and conditions. Detectability depends on input amplitude and output firing rate, and excitatory inputs are detected more readily than inhibitory (Figure 1). Moreover, as we model increasing numbers of presynaptic inputs, we are able to estimate connection strengths more accurately and detect the presence of connections more quickly. We find that model-based methods outperform previous methods for detecting synaptic connections (nonparametric tests on the cross-correlation), and simulation results (adaptive exponential integrate-and-fire) suggest that the model results hold even when synaptic input is conductance-based rather than current-based. Together these results illustrate the possibilities and outline the limits of inferring synaptic input from spikes.

